# Identification of Allergenic Proteins in Velvet Mesquite (*Prosopis velutina*) Pollen: An Immunoproteomics Approach

**DOI:** 10.3390/life12091421

**Published:** 2022-09-13

**Authors:** José Ángel Huerta-Ocampo, Lino Gerardo Batista-Roche, Martha Beatriz Morales-Amparano, María del Refugio Robles-Burgueño, Gabriela Ramos-Clamont Montfort, Luz Vázquez-Moreno, Fernando Ramírez-Jiménez, Luis M. Terán

**Affiliations:** 1 Consejo Nacional de Ciencia y Tecnología, Mexico City 03940, Mexico; 2 Centro de Investigación en Alimentación y Desarrollo, A.C. Hermosillo, Hermosillo 83304, Mexico; 3 Instituto Nacional de Enfermedades Respiratorias “Ismael Cosío Villegas”, Mexico City 14080, Mexico

**Keywords:** allergy, mass spectrometry, mesquite, pollen, two-dimensional gel electrophoresis

## Abstract

Velvet mesquite (*Prosopis velutina*) is a native legume of the southwestern United States and northwestern Mexico, contributing significantly to the desert ecosystem and playing key ecological roles. It is also an important cause of allergic respiratory disease widely distributed in the Sonoran, Chihuahuan, and Mojave Deserts. However, no allergens from velvet mesquite pollen have been identified to date. Pollen proteins were extracted and analyzed by one- and two-dimensional electrophoresis and immunoblotting using a pool of 11 sera from mesquite-sensitive patients as the primary antibody. IgE-recognized protein spots were identified by mass spectrometry and bioinformatics analysis. Twenty-four unique proteins, including proteins well known as pollen, food, airway, or contact allergens and four proteins not previously reported as pollen allergens, were identified. This is the first report on allergenic proteins in velvet mesquite pollen. These findings will contribute to the development of specific diagnosis and treatment of mesquite pollen allergy.

## 1. Introduction

*Prosopis* spp. are members of a genus with more than 40 species that belong to the Fabaceae family and are widely distributed in the desert and semi-desert regions of Africa, Asia, and America. Some species within the genus are invasive and potentially harmful in the ecosystems where they are introduced [1]. Whereas *P. juliflora* is found in almost all of Mexico, mainly in arid places, from Baja California and Chihuahua to Oaxaca, and from Tamaulipas to Veracruz, *P. velutina* (velvet mesquite) is a legume native to the Sonoran, Chihuahuan, and Mojave Deserts (northwestern Mexico and the southwestern United States) where it plays key ecological roles, contributing significantly to the desert ecosystem to which this plant species is restricted [2,3]. Tree pollens are the most abundant in Mexico, outnumbering weed and grass pollens [4]. Positivity to mesquite pollen in allergic patients is high (21%) [5]. In addition, mesquite pollen represents an important source of pollinosis in cities of the Sonoran Desert such as Hermosillo [6,7].

Some studies reported the immunodetection of IgE-binding proteins with molecular weights between 10 and 99 kDa in *P. juliflora* pollen [1]. Additionally, only two mesquite allergens (Pro j 1, Pro j 2) have been identified, characterized, and officially included in the allergen.org database by the IUIS committee [2,5,8]. However, no velvet mesquite pollen allergens have been identified so far. Interestingly, the Mexican Immunotherapy Guide is considering including Prosopis pollen in the set of skin tests [9]. The scarce number of identified and characterized mesquite pollen proteins constitutes a limitation for the development of diagnostic and immunotherapy strategies [10]. Thus, the aim of this work was to immunodetect, for the first time, the allergenic proteins of velvet mesquite pollen using two-dimensional Western blot analysis and to identify them by tandem mass spectrometry.

## 2. Materials and Methods

### 2.1. Selection of Patients and Healthy Volunteers

Eleven polysensitized patients reactive to mesquite pollen and 4 non-allergic subjects were recruited by the allergy clinic of the National Institute of Respiratory Diseases (INER), Mexico. Skin prick test was performed on the forearm by applying a drop of a commercial allergenic extract (Hollister Stier, Elkhart, IN, USA) with the most common aeroallergens (including mesquite). Subjects with wheal diameter ≥ 3 mm after 20 min of exposure to the extract were considered positive for the test. Histamine (10 mg/mL) was used as positive control and saline solution as negative control. Serum and anticoagulated whole blood collection (for positive and negative patients) was approved by the Ethics and Research Committees of INER, and the study was conducted under the ethical principles of the 1975 Declaration of Helsinki (as revised in 1983), and it was consistent with Good Clinical Practice Guidelines. None of the patients at the time of sampling was receiving immunotherapy, corticosteroids, or antihistamine treatment. Peripheral blood samples were obtained by venipuncture. Anticoagulated whole blood samples were used to estimate the number of leukocytes by using a cell counter (Sysmex XP-300, Sysmex Corporation, Kobe, Japan), and the percentage of eosinophils was estimated by direct observation through Wright’s stain blood preparations. Serum samples were stored at −80 °C until use.

### 2.2. Pollen Collection and Protein Extraction

Inflorescences were collected from velvet mesquite trees (May 2020) in the city of Hermosillo, Sonora, Mexico. Trees were identified as *Prosopis velutina*, according to the dichotomous key reported by Palacios [3]. Anthers were removed from the dry inflorescences using a 2 mm mesh. Pollen grains were separated from the anthers with a 63 μM mesh (VWR International, Radnor, Pennsylvania, USA). Pollen was weighed, and 100 mg of pollen was transferred to 2 mL polypropylene tubes. The tubes were immersed in liquid nitrogen, and the frozen pollen was stored at −80 °C until use. Soluble proteins were extracted according to the method reported previously for *Ligustrum lucidum* pollen [11] with slight modifications. Briefly, 100 mg of pollen was mixed with 3 mL of extraction solution (sucrose, 30%, sodium dodecyl sulphate (SDS), 2%, Tris-HCl, 0.1 M, β-mercaptoethanol, 2%, phenylmethylsulfonyl fluoride, 1 mM), sonicated three times for 1 min at an amplitude of 30% (GE-505 Ultrasonic Processor, Sonics & Materials, Inc., Newtown, CO, USA), then shaken on ice for 10 min at 120 rpm. Phenol solution (equilibrated with 10 mM Tris-HCl, 1 mM EDTA, pH 8.0) was added to the above, mixed, and centrifuged at 5000 rpm/15 min/4 °C. Phenolic phase was recovered and mixed with 100 mM ammonium acetate and incubated overnight at −20 °C. Samples were then centrifuged at 13,000 rpm/15 min/4 °C, the pellet was washed once with cold absolute acetone, and twice with 80% acetone. Pellet was vacuum dried at room temperature and suspended in 600 μL of rehydration solution (8 M urea, 2% 3-((3-cholamidopropyl)dimethylammonio)-1-propanesulfonate, 20 mM dithiothreitol). The resulting protein solution was desalted using a PD-10 column (Cytiva, Uppsala, Sweden), precipitated again with ammonium acetate, centrifuged, and suspended in 600 μL of rehydration solution. Pollen protein concentration was estimated with the RC DC Protein Assay (Bio-Rad, Hercules, CA, USA).

### 2.3. SDS-PAGE and Two-Dimensional Gel Electrophoresis (2-DE)

Pollen soluble proteins (20 µg) were separated by SDS-PAGE and stained with Coomassie blue. For 2-DE, pollen proteins (1.3 mg) were loaded onto 13 cm IPG strips, pH 4–7 (Cytiva, Uppsala, Sweden). Passive rehydration, isoelectric focusing, and protein separation were conducted as reported previously [12]. Preparative gels were stained with Coomassie blue, digitized (Typhoon FLA-9500, GE-Healthcare Bio-Sciences, Uppsala, Sweden), and images were analyzed with PDQuest v8.0.1 (Bio-Rad). Proteins from analytical gels were transferred to a polyvinylidene difluoride (PVDF) membrane (Immun-Blot, Bio-Rad) using a semi-dry transfer system (Hoefer TE77XP, Thermo-Fisher Scientific, Waltham, MA, USA). Membranes were washed with PBS pH 7.5 and blocked with sodium-azide-free casein (0.5%) at 50 rpm and 4 °C overnight.

### 2.4. Immunodetection Analysis

Western blot tests were performed to immunodetect velvet mesquite pollen proteins using serum IgE from allergic patients. PVDF membranes were incubated overnight at 4 °C with individual sera from patients and controls diluted 1:10 in PBS with 0.5% sodium-azide-free casein for one-dimensional Western blot, whereas pooled sera were diluted 1:20 in PBS with 0.5% sodium-azide-free casein for two-dimensional Western blot. After washing (5 times for 5 min with PBS-Tween 20), membranes were incubated with a monoclonal anti-human IgE antibody conjugated to peroxidase diluted to 1 mg/mL in PBS (mouse monoclonal (B3102E8) anti-human IgE Fc (HRP), ABCAM Laboratories, Cambridge, MA, USA) for 2 h at room temperature. Membranes were washed again (4 times for 5 min with PBS-Tween and 1 time with PBS for 5 min). Chemiluminescent development of the membranes was carried out with the Clarity Western ECL substrate (Bio-Rad) and imaged (Chemidoc MP, Bio-Rad). Exposition parameters were automatically optimized by Image-Lab software (Bio-Rad).

### 2.5. In-Gel Protein Digestion and Mass Spectrometry Analysis

Immunodetected spots were cut from preparative 2-DE gels, reduced with 10 mM dithiothreitol, and alkylated with 55 mM iodoacetamide. Proteins were digested with mass-spectrometry-grade trypsin (Pierce Biotechnology, Rockford, IL, USA) overnight at 37 °C and tryptic peptides desalted using C18 Zip-Tips (Waters, Milford, MA, USA). Chromatographic separation of tryptic peptides was performed using the 1290 Infinity LC System (Agilent Technologies, Santa Clara, CA, USA) equipped with a ZORBAX 300SB-C8 column (5 µm × 2.1 mm × 150 mm, Agilent Technologies), and analysis was performed using the Agilent 6530 Q-TOF mass spectrometer, as reported previously [13].

### 2.6. Protein Identification

Mass spectrometry data were searched against the *Prosopis alba* subset of the NCBInr protein database (57,575 sequences, October 2021) using the Spectrum Mill MS Proteomics Workbench server (Agilent Technologies, Santa Clara, CA, USA). Trypsin was used as the specific protease, and one missed cleavage was allowed. Mass error tolerance for precursor and fragment ions was set at 20 ppm and 0.1 Da, respectively. Methionine oxidation and asparagine deamidation were specified as variable modifications, while cysteine carbamidomethylation was specified as fixed modification. Two good peptides (individual peptide ion score > 9 and scored peak intensity ≥ 60) and a protein score > 24 were needed for confident protein identifications.

## 3. Results

### 3.1. Patients

Clinical features of the allergic patients (four with allergic rhinitis, two with asthma, five with asthma + allergic rhinitis) and the control patients (four non-atopic subjects) that participated in the study are shown in Table 1.

### 3.2. Pollen Protein Profiles and Immunodetection of Velvet Mesquite Allergens

**Figure 1:**
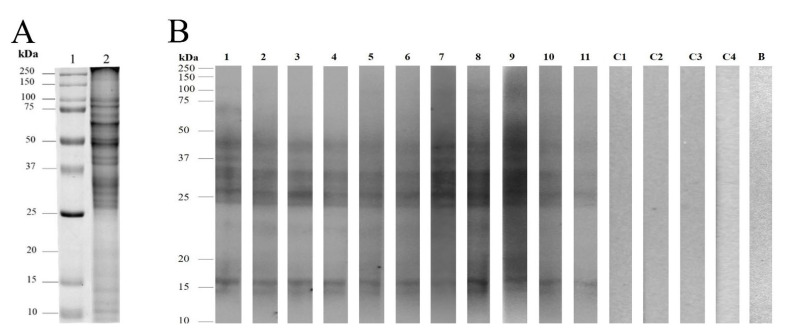
Immunoscreening of the allergens of velvet mesquite (*Prosopis velutina*) pollen grains. (**A**) One-dimensional electrophoresis of *Prosopis velutina* pollen proteins. Lane 1, molecular weight marker. Lane 2, total soluble pollen proteins (20 μg). (**B**) Western blot showing IgE-based immunodetection of protein bands in 11 atopic patient sera. Lanes C1–C4, negative control blots with individual serum from four healthy volunteers. Lane B, reagent blank (without primary antibody).

Pollen protein extraction (100 mg starting material) yielded 87.5 mg/g of pollen. The SDS-PAGE profile showed that the most intense bands migrated between 25 and 100 kDa (Figure 1A); one-dimensional immunodetection using individual sera from allergic patients revealed nine major IgE-binding protein bands (14.5; 16; 24.5; 26; 33; 36; 41; 44; 81 kDa), since they were immunodetected in more than 80% of the patients. In contrast, sera from healthy volunteers did not exhibit any IgE-binding reactivity (Figure 1B).

**Figure 2:**
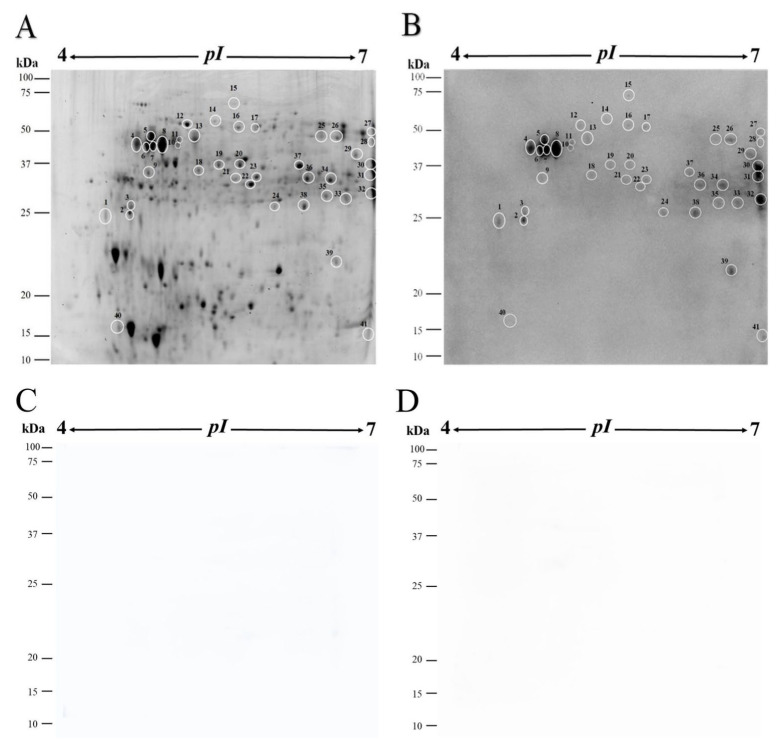
2-DE and immunoblotting using pollen proteins from velvet mesquite. (**A**) 2-DE profile of total soluble proteins stained with Coomassie blue. (**B**) Protein spots detected with pooled sera from 11 allergic patients using 2-DE immunoblotting. (**C**) Reagent control (without primary antibody). (**D**) Negative control (pooled serum samples from four healthy volunteers).

The 2-DE profile was resolved into 652 spots (Figure 2A). Protein spots were in the 10–100 kDa range of molecular masses and pH range of 4–7. The 2D Western blot revealed 41 spots recognized by serum IgE antibodies from allergic patients. Immunodetected spots were found between 14.5 and 75 kDa and isoelectric points from 4.5 to 6.9 (Figure 2B). Only five spots (spots 1, 2, 40, 39, and 41) with molecular masses below 25 kDa were immunodetected.

### 3.3. Protein Identification

A database search using Spectrum Mill MS Proteomics Workbench server Version B.06.00 (Agilent Technologies, Santa Clara, CA, USA), and mass spectrometry data allowed the identification of proteins in 36 of 41 immunodetected spots (Table 2). Whereas three spots (3, 10, and 15) were not identified, spots 8 and 11 presented excellent peptides (one-hit wonders) with scores of 17.25 and 20.89, respectively, corresponding to a polygalacturonase-like protein. Interestingly, 24 unique proteins were identified among the protein spots. Immunodetected proteoforms included triosephosphate isomerase (*n* = 2); polygalacturonase (*n* = 6); enolase (*n* = 3); glutelin type-D 1 (*n* = 2); UDP-arabinopyranose mutase 1 (*n* = 2); S-adenosylmethionine synthase 1 (*n* = 2); and glyceraldehyde-3-phosphate dehydrogenase (*n* = 2) (Table 2, Supplementary Materials Table S1).

## 4. Discussion

Mesquite pollen allergy is clinically relevant worldwide, particularly in desert and semi-desert regions. In addition, the influence of climate change and environmental pollution is expected to increase the allergenicity of this pollen [2]. To date, only two mesquite allergens have been identified and characterized. The allergens Pro j 2 [8] and Pro j 1 from *P. juliflora* [14] have been recombinantly expressed and characterized. In this study, we identified for the first time velvet mesquite pollen allergenic proteins using an immunoproteomics discovery approach (Table 2).

Polygalacturonases are major pollen allergens in Cupressaceae trees [15]. Additionally, a polygalacturonase was purified and identified as a major allergen (Pla a 2) in *Platanus acerifolia* pollen [16]. Glucan endo-1, 3-beta-glucosidase (spot 32), has been reported as allergenic in peach tree pollen [17]. Fructokinases (spot 22) have been identified as allergenic in coconut and pecan pollen [18,19]. We also identified two proteoforms of UDP-arabinopyranose mutase 1 (spots 19 and 20) in velvet mesquite pollen. This enzyme was recently reported as an allergen in *Delonix regia* pollen [20].

Two proteoforms of triosephosphate isomerase were immunodetected (spots 24 and 38) in this study, similar to those reported in pecan pollen and latex [18,21]. Pyruvate dehydrogenase E1 component subunit beta, mitochondrial (spot 18) was, for the first time, immunodetected in pollen protein using the serum from allergic patients. We also identified three enolase proteoforms (spots 17, 21, and 23). Enolases are highly conserved among organisms from different taxonomical groups. Enolases derived from diverse sources (fish, fungi, pollens, and latex) have been extensively investigated and associated with allergic diseases [22].

Glyceraldehyde-3-phosphate dehydrogenase (spots 9, 31, and 35) has been previously described as allergenic in pecan and sunflower pollen [18,23]. Uridine triphosphate-glucose-1-phosphate uridylyltransferase (spot 16) was also immunodetected in peach and red oak pollen [17,24]. Fructose-bisphosphate aldolase (spot 30) is allergenic in pecan and cashew tree pollen [18,25]. Mitochondrial malate dehydrogenase (spot 37) has been reported as allergenic in Senecio and olive pollen [26,27]. The major allergen Mal f 4 of yeast *Malassezia furfur*, which causes atopic dermatitis in allergic patients, is also a mitochondrial malate dehydrogenase [28].

Thaumatin-like protein (spot 1) belongs to the pathogenesis-related-5 family. Thaumatin-like proteins are important pollen (e.g., cypress, cedar, olea) and food (e.g., kiwi, chili, apple, banana, peach, and cherry) allergens [29,30]. Two proteins were identified in spot 41: nucleoside diphosphate kinase, which has been reported as an allergen in largemouth bass [31] and was reported for the first time as a pollen protein recognized by IgE in pecan nut [18], and pathogenesis-related protein 1, which has been previously identified as a pollen (Bermuda grass, wormwood and mugwort) and food (Muskmelon) allergen [32,33].

Proteasome subunit alpha (spot 33) was immunodetected as allergenic in corn flour and latex [34,35]. Mitochondrial-processing peptidase subunit alpha (spot 14) was identified for the first time as IgE-recognized in the present work. GDSL esterase/lipase (spot 29) was reported as allergenic in cashew tree pollen [25]. Manganese superoxide dismutase (spot 39) is known as a contact allergen in *Hevea brasiliensis* latex, as a food allergen (pistachio nut), and as an airway allergen from fungi (*Alternaria, Aspergillus*) [36,37,38,39]. It was also reported as allergenic in grass pollen [40]. The ATP synthase subunit beta (spot 12) has been reported to be allergenic in pollen from date palm, red oak, and gulmohar tree (among others) [20,24,41].

Rho GDP dissociation inhibitor 1 (spot 2) and glutelin type-D 1 (spots 34 and 36) have not been reported as pollen allergens before. However, the allergenic capacity of glutelin-type proteins was verified by immunodetection of recombinant proteins obtained from buckwheat seeds [42]. Carbonic anhydrase (spot 13) has been recognized by IgE from patients allergic to palm and pecan pollen [18,41].

S-adenosylmethionine synthase (spots 25 and 26) was identified as an IgE-binding protein in coconut pollen and gulmohar tree [19,20]. Profilin (spot 40) has previously been identified as an allergen in *Prosopis juliflora* and was named Pro j 2 [8]. Allergenic profilins are reported in various pollens, including olive, London planetree, amaranth, and sugar beet, among many others and are considered clinically relevant panallergens, showing extensive cross-reactivity between pollens and other foods of plant origin. Furthermore, the cross-reaction of pollen and fruit profilins can trigger oral allergy syndrome in allergic patients [43,44].

## 5. Conclusions

We used an immunoproteomics approach to uncover velvet mesquite pollen allergens. This represents the first report for allergenic proteins in velvet mesquite pollen. We identified 24 unique proteins, comprising proteins extensively recognized as pollen allergens and panallergens. Interestingly, four unique identified proteins have not been reported before as pollen allergens. However, more studies (Western blot/ELISA) using purified natural or recombinant proteins using patient’s serum are necessary to irrefutably confirm IgE reactivity in these four new, suspected allergens. These findings may contribute to the development of immunotherapeutic strategies for respiratory allergies, comprising the production of recombinant versions of these allergenic proteins with applications also in the clinic as diagnostic agents and to replace the use of whole pollen extracts, thus, eliminating unwanted side effects and making allergen immunotherapy safer and diagnosis more specific.

## Figures and Tables

**Table 1:** Clinical characteristics of allergic and non-atopic subjects.

	Allergic Patients	Control Subjects
No. subjects	11	4
Age (years)	42 ± 3.7	36 ± 3.0
Females	7	3
Males	4	1
FEV_1_%	85% (81–93)	104% (98–113) *
Total IgE (U/dL)	330 (90–1480)	60 (25–84) *
Eosinophils (cells/mm^3^)	335 (190–680)	105 (59–140) *
Atopy	yes	no
Asthma	2	0
Asthma + Allergic rhinitis	5	0
Allergic rhinitis	4	0

FEV1 = forced expiratory volume at the end of the 1st second. * *p* < 0.05. Statistical analysis by Mann–Whitney U test.

**Table 2:** Allergenic proteins identified in velvet mesquite (*Prosopis velutina*) pollen.

Protein	Accession Number	Mr/*pI* Theor. ^‡^	PM/SC ^§^	Score ^¶^	Spot
ATP synthase subunit beta, mitochondrial	1624089683	60.1/5.90	19/43.6%	378.49	12
Carbonic anhydrase	1624114052	37.3/8.79	2/7.2%	34.47	13
Enolase	1624040022	48.0/5.83	7/18.4%	121.01	17
Enolase	1624040022	48.0/5.83	7/17.7%	120.93	23
Enolase	1624040022	48.0/5.83	5/12.3%	87.65	21
Fructose-bisphosphate aldolase	1624108864	38.8/6.69	13/38.5%	242.47	30
GDSL esterase/lipase	1624029014	37.9/6.07	7/16.7%	101.4	29
Glucan endo-1,3-beta-glucosidase	1624040970	38.9/8.81	5/18.6%	92.67	32
Glutelin type-D 1	1624065233	38.4/6.33	7/20.5%	121.26	36
Glutelin type-D 1	1624065233	38.4/6.33	5/14.3%	85.23	34
Glyceraldehyde-3-phosphate dehydrogenase	1624127796	36.5/7.81	12/43.9%	183.42	31
Glyceraldehyde-3-phosphate dehydrogenase	1624127796	36.5/7.81	11/37.3%	182.95	35
Glyceraldehyde-3-phosphate dehydrogenase	1624102601	36.9/8.92	2/4.7%	24.3	9
Malate dehydrogenase, mitochondrial	1624051156	36.3/8.81	10/30.7%	188.26	37
Mitochondrial-processing peptidase subunit α	1624112156	55.2/5.87	6/13.2%	79.45	14
Nucleoside diphosphate kinase	1624076552	16.4/6.85	6/39.8%	109.08	41
Pathogenesis-related protein 1	1624126639	18.1/9.05	2/17.2%	30.44	41
Polygalacturonase-like	1624065841	38.4/6.32	5/15.3%	83.14	27
Polygalacturonase-like	1624065851	43.6/6.72	5/14.5%	73.17	28
Polygalacturonase-like	1624065720	42.0/5.18	3/10.7%	63.09	6
Polygalacturonase-like	1624065720	42.0/5.18	3/10.7%	56.24	4
Polygalacturonase-like	1624065720	42.0/5.18	2/7.3%	41.01	5
Polygalacturonase-like	1624065720	42.0/5.18	2/7.3%	29.11	7
Polygalacturonase-like *	1624023427	42.2/5.20	1/3.3%	20.89	11
Polygalacturonase-like *	1624023427	42.5/5.20	1/3.3%	17.25	8
Probable fructokinase-5	1624052972	35.3/5.79	13/44.3%	234.55	22
Profilin	1624112235	14.4/4.78	3/25.5%	51.28	40
Proteasome subunit alpha	1624127659	27.5/6.75	6/26.4%	109.34	33
Pyruvate dehydrogenase E1 component subunit beta	1624021117	40.2/5.77	4/11.1%	65.55	18
Rho GDP dissociation inhibitor 1	1624023092	25.2/4.70	2/16.5%	27.99	2
S-adenosylmethionine synthase	1624121540	43.0/6.08	4/12.5%	75.79	26
S-adenosylmethionine synthase	1624121540	43.0/6.08	3/11.7%	47.09	25
Superoxide dismutase, mitochondrial	1624128322	26.5/7.20	4/18.8%	62.58	39
Thaumatin-like protein 1b	1624039760	25.8/5.05	2/9.9%	32.13	1
Triosephosphate isomerase, cytosolic	1624021851	27.2/5.88	9/51.9%	168.05	38
Triosephosphate isomerase, cytosolic	1624021851	27.2/5.88	4/16.2%	80.16	24
UDP-arabinopyranose mutase 1	1624085496	41.1/5.65	11/28.4%	187.21	20
UDP-arabinopyranose mutase 1	1624085496	41.1/5.65	10/27%	157.58	19
UTP-glucose-1-phosphate uridylyltransferase	1624020612	51.9/5.6	15/34.1%	273.56	16

**^‡^** Theoretical molecular mass (kDa)/isoelectric point; **^§^** Number of peptides matched/protein sequence coverage. ^¶^ Spectrum Mill protein score (scores ≥ 24 and at least two peptides were necessary for confident protein identification). * Proteins putatively identified by one outstanding-scoring peptide.

## Data Availability

Data availability upon request.

## Supplementary Materials

The following supporting information can be downloaded at: https://www.mdpi.com/article/10.3390/life12091421/s1, Table S1: Allergenic proteins identified in velvet mesquite (*Prosopis velutina*) pollen by tandem mass spectrometry and bioinformatic analysis.
