# Supplementary material for: Identification of Allergenic Proteins in Velvet Mesquite (Prosopis velutina) Pollen: An Immunoproteomics Approach
Source: Life (Basel). 2022 Sep 13;12(9):1421. doi: 10.3390/life12091421 (PMC9502229; doi:10.3390/life12091421)
Supplement: Supplementary file 1 [file life-12-01421-s001.zip › life-1887562-supplementary.pdf]

Supplementary files

Identification of allergenic proteins in velvet mesquite (*Prosopis velutina*) pollen: an immunoproteomics approach

José Ángel Huerta-Ocampo [a,b*], Lino Gerardo Batista-Roche [b], Martha Beatriz Morales-Amparano [b], María del Refugio Robles-Burgueño [b], Gabriela Ramos-Clamont Montfort [b], Luz Vázquez-Moreno [b], Fernando Ramírez-Jiménez [c], Luis M. Terán [c,*].

[a] *Consejo Nacional de Ciencia y Tecnología, Mexico City, 03940, Mexico*

[b] *Centro de Investigación en Alimentación y Desarrollo, A.C. Hermosillo, Sonora 83304, México*

[c] *Instituto Nacional de Enfermedades Respiratorias "Ismael Cosío Villegas", Ciudad de México 14080, México*

* *Corresponding authors: E-mail*: jose.huerta@ciad.mx (J.A. Huerta-Ocampo) +52 6622892400, Ext 513. lmteran@iner.gob.mx (L.M. Terán) +52 5554871700, Ext.5259.

Supplementary Table S1. Allergenic proteins identified in velvet mesquite (*Prosopis velutina*) pollen

Spot	Protein	Accession number[a]	Mr/*pI* Exp.[b]	Mr/*pI* Theor.[c]	PM/SC[d]	Score[e]	Peptide Sequences
1	Thaumatin-like protein 1b	1624039760	24.8/4.51	25.8/5.05	2/9.9%	32.13	(K)ANINAACPNDLK(V) + Deamidated N<br>(K)VTGSDGNVIACK(S)
2	Rho GDP-dissociation inhibitor 1-like	1624023092	25.0/4.74	25.2/4.70	2/16.5%	27.99	(R)HASESSVAPTEDDDDDEGTKLELGPK(C)<br>(K)VLSLSIISPGR(D)
3	Not identified	----------------	--------------	-----------------	------------	----------	--
4	Polygalacturonase-like	1624065720	44.9/4.80	42.0/5.18	3/10.7%	56.24	(K)AWTDACAATEASK(V)<br>(K)VLVGAGTYNMNAVDLK(G)<br>(R)YNNEQPVSNVNVK(N)
5	Polygalacturonase-like	1624065720	47.8/4.93	42.0/5.18	2/7.3%	41.01	(K)VLVGAGTYNMNAVDLK(G)<br>(R)YNNEQPVSNVNVK(N)
6	Polygalacturonase-like	1624065720	43.9/4.90	42.0/5.18	3/10.7%	63.09	(K)AWTDACAATEASK(V)<br>(K)VLVGAGTYNMNAVDLK(G)<br>(R)YNNEQPVSNVNVK(N)
7	Polygalacturonase-like	1624065720	43.9/4.95	42.0/5.18	2/7.3%	29.11	(K)VLVGAGTYNMNAVDLK(G)<br>(R)YNNEQPVSNVNVK(N)
8	Polygalacturonase-like*	1624023427	44.6/5.03	42.5/5.20	1/3.3%	17.25	(R)YDNEAPVSNVNVK(N)
9	Glyceraldehyde-3-phosphate dehydrogenase 2, cytosolic-like isoform X1	1624102601	34.6/4.91	36.9/8.92	2/4.7%	24.3	(K)IGINGFGR(I) + Deamidated N<br>(K)VLPALNGK(L)
10	Not identified	----------------	--------------	-----------------	------------	----------	--
11	Polygalacturonase-like*	1624023427	47.1/5.19	42.2/5.20	1/3.3%	20.89	(R)YDNEAPVSNVNVK (N)
12	ATP synthase subunit beta, mitochondrial	1624089683	53.6/5.27	60.1/5.90	19/43.6 %	378.49	(K)AHGGFSVFAGVGER(T)<br>(R)DAEGQDVLLFIDNIFR(F)<br>(R)EAPAFVEQATEQQILVTGIK(V)<br>(R)EGNDLYR(E)<br>(R)EMIESGVIK(L)<br>(R)FTQANSEVSALLGR(I)<br>(K)GAIGQVCQVIGAVVDVR(F)<br>(K)IGLFGGAGVGK(T)<br>(R)IINVIGEAIDEK(G)<br>(R)IPSAVGYQPTLATDLGGLQER(I)<br>(K)IVDEFTGK(G)<br>(K)KDPGTGGGK(I)<br>(K)LGDKQSESK(C)<br>(R)LVLEVAQHLGENMVR(T) + Oxidized M<br>(R)TIAMDGTEGVVR(G) + Oxidized M<br>(K)TVLIMELINNVAK(A)<br>(R)VGLTGLTVAEHFR(D)<br>(R)VLNTGSPITVPVGR(A)<br>(K)VVDLLAPYQR(G)
13	Carbonic anhydrase, chloroplastic isoform X1	1624114052	47.1/5.34	37.3/8.79	2/7.2%	34.47	(K)EAVNVSLGNLLTYPFVR(E)<br>(K)GGYYDFVK(G)
14	Mitochondrial-processing peptidase subunit alpha-like	1624112156	56.5/5.51	55.2/5.87	6/13.2%	79.45	(K)AVDEVTLKDITSISQK(L)<br>(R)EVEAIGGNVQASASR(E)<br>(K)ITTLPNGVK(V) + Deamidated N<br>(R)KPVESFLK(A)<br>(R)MVASEDIGR(Q)<br>(K)SAILMNLESR(M) + Oxidized M
15	Not identified	----------------	--------------	-----------------	------------	----------	--

16	UTP--glucose-1-phosphate uridylyltransferase-like	1624020612	52.4/5.74	51.9/5.6	15/34.1 %	273.56	(R)ANPANPAIELGPEFK(K)<br>(R)ANPANPAIELGPEFKK(V)<br>(R)DGLTFLDLIVK(Q)<br>(K)DGVKLEVPDGAK(I)<br>(R)FFDNAIGINVPR(S)<br>(K)IATPTDEVVVPYDSLAPSPQDS SEIKK(L)<br>(K)ILNHLVQK(K)<br>(K)LEVPDGAK(I)<br>(K)LLDKLAVLK(L)<br>(R)LVEADALK(M)<br>(R)LVEADALKMEIIPNPK(E) + Oxidized M<br>(R)LVVDDYLPLPSK(G)<br>(K)SAVAELSQISENEK(N)<br>(K)SIPSIVELDNLK(V)<br>(K)VLQLETAAGAAIR(F)
17	Enolase	1624040022	52.4/5.90	48.0/5.83	7/18.4%	121.01	(R)DGGSDYLGK(G)<br>(K)EGLELLK(T)<br>(R)IEEELGSDAVYAGASFR(A)<br>(K)IIAPALVGK(D)<br>(K)ISGDALKDLYK(S)<br>(K)VNQIGSVTESIEAVR(M)<br>(K)VQIVGDDLLVTNPK(R)
18	Pyruvate dehydrogenase E1 component subunit beta, mitochondrial	1624021117	35.3/5.37	40.2/5.77	4/11.1%	65.55	(K)EGMSAEVINLR(S)<br>(R)MAVPQVEDIVR(A)<br>(R)STINASVR(K)<br>(K)VLSPYSSEDAR(G)
19	UDP-arabinopyranose mutase 1	1624085496	36.6/5.55	41.1/5.65	10/27%	157.58	(K)ASCISFK(D)<br>(K)ASNPFVNLR(K)<br>(R)GYPFSLR(E)<br>(K)INALEQHIK(N)<br>(K)TGLPYIYHSK(A)<br>(K)TISVPDGFDYELYNR(N)<br>(K)VICDHLGLGVK(T)<br>(K)YIYTIDDDCFVASDPSGK(K)<br>(K)YIYTIDDDCFVASDPSGKK(I)<br>(R)YVDAVLTIPK(G)
20	UDP-arabinopyranose mutase 1	1624085496	36.6/5.75	41.1/5.65	11/28.4 %	187.21	(K)ASCISFK(D)<br>(K)ASNPFVNLR(K)<br>(K)ASNPFVNLRK(E)<br>(R)GYPFSLR(E)<br>(K)INALEQHIK(N)<br>(K)TGLPYIYHSK(A)<br>(K)TISVPDGFDYELYNR(N)<br>(K)TISVPDGFDYELYNRNDINR(I)<br>(K)VICDHLGLGVK(T)<br>(K)YIYTIDDDCFVASDPSGK(K)<br>(R)YVDAVLTIPK(G)
21	Enolase	1624040022	33.1/5.71	48.0/5.83	5/12.3%	87.65	(K)ACNALLLK(V)<br>(K)EGLELLK(T)<br>(K)ISGDALKDLYK(S)<br>(K)VNQIGSVTESIEAVR(M)<br>(K)VQIVGDDLLVTNPK(R)
22	Probable fructokinase-5	1624052972	33.6/5.85	35.3/5.79	13/44.3 %	234.55	(K)APGGAPANVACAIAK(L)<br>(R)EAGALLSYDPNVR(L)<br>(K)EALTFANACAALCTTQK(G)<br>(K)GAIPALPTASDAQSLISK(S)<br>(K)IFHYGSISLISEPCR(S)<br>(K)LGGNSAFIGK(V)<br>(K)LLIVTDGEK(G)<br>(R)MLADILK(K)<br>(R)MLADILKK(N)<br>(K)NGVNTDGVCFDADAR(T)<br>(R)SAHMAAMK(A)<br>(R)TALAFVTLR(N)<br>(K)VGDDEFGR(M)
23	Enolase	1624040022	36.6/5.90	48.0/5.83	7/17.7%	120.93	(K)ACNALLLK(V)<br>(K)EGLELLK(T)<br>(K)ISGDALKDLYK(S)<br>(K)MGVEVYHHLK(A)<br>(K)VNQIGSVTESIEAVR(M)<br>(K)VQIVGDDLLVTNPK(R)<br>(K)VVIGMDVAASEFYK(S)

24	Triosephosphate isomerase, cytosolic	1624021851	26.3/6.07	27.2/5.88	4/16.2%	80.16	(R)IIYGGSVNGANCK(E) + Deamidated N<br>(K)VATPAQAQEVHAELR(K)<br>(K)VATPAQAQEVHAELRK(W)<br>(K)VIACVGETLEQR(E)
25	S-adenosylmethionine synthase 1-like	1624121540	47.8/6.50	43.0/6.08	3/11.7%	47.09	(R)FVIGGPHGDAGLTGR(K)<br>(K)SVVASGLAR(R)<br>(K)VLVNIEQQSPDIAQGVHGHLTK(K)
26	S-adenosylmethionine synthase 1-like	1624121540	47.1/6.65	43.0/6.08	4/12.5%	75.79	(R)FVIGGPHGDAGLTGR(K)<br>(R)GIGFVSADVGLDADNCK(V)<br>(K)SVVASGLAR(R)<br>(K)VACETCTK(T)
27	Polygalacturonase-like	1624065841	50.0/6.97	38.4/6.32	5/15.3%	83.14	(R)FDNVDIK(Y)<br>(K)IQISDVTFR(N)<br>(K)STNVNVINSR(I)<br>(K)TPILIDQEYCPSK(K)<br>(K)VDGTIQGIINPSTLK(Q)
28	Polygalacturonase-like	1624065851	45.7/6.98	43.6/6.72	5/14.5%	73.17	(K)FVEETPIDKFTVK(N)<br>(K)GLPNGDITQALTK(A) + Deamidated N<br>(K)ISDVTFK(N)<br>(K)VLDIASYK(G)<br>(K)VSAPEDSPNTDGIHVGR(S)
29	GDSL esterase/lipase At1g29670-like	1624029014	40.6/6.82	37.9/6.07	7/16.7%	101.4	(K)AAFLFNANLR(S)<br>(R)GVNYASGAAGIR(N)<br>(K)HLGSDISLR(L)<br>(K)IDPSLGFK(V)<br>(R)LQLTNHR(T)<br>(K)SSEKVEQLLNK(C)<br>(K)VEQLLNK(C)
30	Fructose-bisphosphate aldolase 6, cytosolic-like isoform X2	1624108864	36.6/6.98	38.8/6.69	13/38.5 %	242.47	(R)ALQQSTIK(A)<br>(K)ANSEATLGTYK(G)<br>(K)CADVTER(V)<br>(K)EGGVLPGIK(V)<br>(K)GILAADESTGTIGK(R)<br>(K)GILAADESTGTIGKR(L)<br>(K)IGPNEPSPLAIHENAYGLAR(Y)<br>(R)LASINVENVESNR(R)<br>(K)TASGKPFVDVLK(E)<br>(R)VAPEVIAEHTVR(A)<br>(R)VLAACYK(A)<br>(K)YQDELIANAAYIGTPGK(G)<br>(K)YYEAGAR(F)
31	Glyceraldehyde-3-phosphate dehydrogenase, cytosolic	1624127796	34.0/6.98	36.5/7.81	12/43.9 %	183.42	(R)AASFNIIPSSTGAAK(A)<br>(K)AGISLSNNFVK(L)<br>(K)DAPMFVVGVNEK(E) + Oxidized M<br>(K)EASEGSLK(G)<br>(K)GASYDEIK(A)<br>(K)GVLGYTEDDVVSTDFVGDCR(S)<br>(K)IGINGFGR(I) + Deamidated N<br>(K)SDINIVSNASCTTNCLAPLAK(V)<br>(K)TLVFGDKPVSVFGTK(N)<br>(K)VIISAPSK(D)<br>(K)VLPSLNGK(L) + Deamidated N<br>(R)VPTVDVSVVDLTVR(L)
32	Glucan endo-1,3-beta-glucosidase, basic isoform-like	1624040970	30.1/6.98	38.9/8.81	5/18.6	92.67	(R)AVQNVYQAIR(A)<br>(R)GYQNLFDALLDSVHAALDSTK(I)<br>(R)LYDPNQAALEALR(N)<br>(R)TYLDNLIR(H)<br>(K)YIAVGNEVNPVGR(N)
33	Proteasome subunit alpha type-4-like	1624127659	28.4/6.73	27.5/6.75	6/26.4	109.34	(K)AAAIGANNQAAQSILK(Q)<br>(K)DGVVLVGEK(K)<br>(K)HGVTQPPTDTA(-)<br>(K)LLQTSTSTEK(M)<br>(R)TTIFSPEGR(L)<br>(K)YQVCSPETLTK(L)
34	Glutelin type-D 1-like	1624065233	32.7/6.59	38.4/6.33	5/14.3%	85.23	(K)AGNLFIVPR(F)<br>(K)ALSPTVLQAGFNVDSK(L)<br>(K)LEQLFR(S)<br>(K)TLVGSQSGK(G)<br>(R)TSDAIFFPPPK(-)

35	Glyceraldehyde-3-phosphate dehydrogenase, cytosolic	1624127796	28.9/6.54	36.5/7.81	11/37.3 %	182.95	(R)AASFNIIPSSTGAAK(A)<br>(K)AGISLSNNFVK(L)<br>(K)DAPMFVVGVNEK(E)<br>(K)EASEGSLK(G)<br>(K)GASYDEIK(A)<br>(K)GVLGYTEDDVVSTDFVGDCR(S)<br>(K)KVIISAPSK(D)<br>(K)SDINIVSNASCTTNCLAPLAK(V)<br>(K)VIISAPSK(D)<br>(K)VLPSLnGK(L) + Deamidated N<br>(R)VPTVDVSVVDLTVR(L)
36	Glutelin type-D 1-like	1624065233	33.1/6.37	38.4/6.33	7/20.5%	121.26	(K)AGNLFIVPR(F)<br>(K)ALSPTVLQAGFNVDSK(L)<br>(R)AQVVGPDGKR(V)<br>(K)LALEKNGFALPR(Y) + Deamidated<br>(K)LEQLFR(S)<br>(K)TLVGSQSGK(G)<br>(R)TSDAIFFPPPK(-)
37	Malate dehydrogenase, mitochondrial	1624051156	36.1/6.30	36.3/8.81	10/30.7 %	188.26	(K)ALEGSDVVIIPAGVPR(K)<br>(K)ANLGDDDIK(A)<br>(R)DDLFNINAGIVK (S)<br>(R)LFGVTTLDVVR(A)<br>(K)QGLESLLPELK(A)<br>(K)RLFGVTTLDVVR(A)<br>(K)RTQDGGTEVVEAK(A)<br>(R)SEVAGYQGEEELGK(A)<br>(R)TQDGGTEVVEAK(A)<br>(K)VAVLGAAGGIGQPLSLLMK(L)
38	Triosephosphate isomerase, cytosolic	1624021851	26.5/6.34	27.2/5.88	9/51.9%	168.05	(R)EAGSTMAVVAEQTK(A) + Oxidized M<br>(K)FFVGGNWK(C)<br>(R)IIYGGSVNGANCK(E) + Deamidated N<br>(K)IVTTLNEAQVPGEDVVEVVVSPPFVFLSLVK(S)<br>(R)QLFNEANEFVADK(V)<br>(K)VATPAQAQEVHAELR(K)<br>(K)VAYALSQGLK(V)<br>(K)VIACVGETLEQR(E)<br>(K)WIHSNVSAEVASSVR(I)
39	Superoxide dismutase [Mn], mitochondrial-like	1624128322	22.0/6.64	26.5/7.20	4/18.8%	62.58	(K)ALEQLHDAMEK(G)<br>(K)HHQTYITNYNK(A)<br>(R)LVVETTANQDPLVTK(G)<br>(K)YASEVYEK(E)
40	Profilin	1624112235	16.1/4.63	14.4/4.78	3/25.5%	51.28	(K)DFEEPGYLAPK(G)<br>(R)LGDYLVDQGL(-)<br>(K)YMVIQGEPGAVIR(G) + Oxidized M
41	Nucleoside diphosphate kinase 1	1624076552	14.5/6.98	16.4/6.85	6/39.8%	109.08	(R)GDYAIDIGR(N)<br>(R)GLVGEIISR(F)<br>(K)IIGATNPAQSEPGTIR(G)<br>(K)LISVDRPFAEK(H)<br>(R)NVIHGSDSVESAR(K)<br>(R)NVIHGSDSVESARK(E)
	Pathogenesis-related protein 1-like	1624126639	14.5/6.98	18.1/9.05	2/17.2%	30.44	(R)LVHSGGDGK(Y)<br>(R)SQVGVPNIAWDETVAAFAR(N)

Spot numbers according to **Fig. 1**. [a] Accession numbers according to the *Prosopis alba* subset of the nrNCBI protein database. [b] Experimental molecular mass (kDa)/isoelectric point. [c] Theoretical molecular mass (kDa)/isoelectric point. [d] Number of peptides matched/protein sequence coverage. [e] Spectrum Mill Protein Score (Scores ≥24 and at least two peptides were necessary for confident protein identification).
